# Connexin hemichannel blockade improves survival of striatal GABA-ergic neurons after global cerebral ischaemia in term-equivalent fetal sheep

**DOI:** 10.1038/s41598-017-06683-1

**Published:** 2017-07-24

**Authors:** Robert Galinsky, Joanne O. Davidson, Christopher A. Lear, Laura Bennet, Colin R. Green, Alistair J. Gunn

**Affiliations:** 1 Department of Physiology, The University of Auckland, Auckland, New Zealand; 2 The Ritchie Centre, Hudson Institute of Medical Research, Victoria, Australia; 3 Department of Ophthalmology, The University of Auckland, Auckland, New Zealand

## Abstract

Basal ganglia injury at term remains a major cause of disability, such as cerebral palsy. In this study we tested the hypotheses that blockade of astrocytic connexin hemichannels with a mimetic peptide would improve survival of striatal phenotypic neurons after global cerebral ischaemia in term-equivalent fetal sheep, and that neuronal survival would be associated with electrophysiological recovery. Fetal sheep (0.85 gestation) were randomly assigned to receive a short or long (1 or 25 h) intracerebroventricular infusion of a mimetic peptide or vehicle, starting 90 minutes after 30 minutes of cerebral ischaemia. Sheep were killed 7 days after ischaemia. Cerebral ischaemia was associated with reduced numbers of calbindin-28k, calretinin, parvalbumin and GAD positive striatal neurons (P < 0.05 ischaemia + vehicle, n = 6 vs. sham ischaemia, n = 6) but not ChAT or nNOS positive neurons. Short infusion of peptide (n = 6) did not significantly improve survival of any striatal phenotype. Long infusion of peptide (n = 6) was associated with increased survival of calbindin-28k, calretinin, parvalbumin and GAD positive neurons (P < 0.05 vs. ischaemia + vehicle). Neurophysiological recovery was associated with improved survival of calbindin-28k, calretinin and parvalbumin positive striatal neurons (P < 0.05 for all). In conclusion, connexin hemichannel blockade after cerebral ischaemia in term-equivalent fetal sheep improves survival of striatal GABA-ergic neurons.

## Introduction

Basal ganglia injury after hypoxia ischaemia (HI) at term remains a major contributor to poor neurodevelopmental outcomes such as cerebral palsy, learning disability and epilepsy^1, 2^. Basal ganglia injury occurs in approximately 25% of cases of encephalopathy at term and is strongly associated with poor neurophysiological outcomes, such as increased severity of neonatal seizures^3^, and discontinuity of electroencephalographic activity^4, 5^. Although therapeutic hypothermia reduced the incidence and severity of injury and improved survival without disability after moderate to severe encephalopathy^6^, nearly half of treated infants still die or survive with disability. Therefore there is a crucial need to identify other therapies to reduce the incidence and severity of hypoxic ischaemic encephalopathy (HIE) at term.

Consistent with clinical HIE, global cerebral ischaemia in term-equivalent fetal sheep, induced by complete bilateral occlusion of the carotid arteries, is associated with profound loss of EEG activity, electrographic seizures and basal ganglia injury^7–9^. Recent work has shown that opening of astrocytic connexin hemichannels (Connexin 43 (Cx43)), the constituents of gap junctions, during ischaemia is implicated in the pathogenesis of HIE^10–12^. Post-insult blockade of Cx43 hemichannels using a mimetic peptide (Peptide 5^13^), targeting the extracellular loop of Cx43 was associated with improved recovery of EEG power, reduced electrographic seizures and improved survival of oligodendrocytes after global cerebral ischaemia, but only an intermediate improvement in survival of cortical neurons^10, 11^. It remains unclear whether the apparent neuroprotective effect of Cx43 hemichannel blockade is selective for particular cell types. To examine this further, we tested the effect of Cx43 hemichannel blockade on the striatum, a major nucleus within the basal ganglia that is highly susceptible to cerebral ischaemia and where neuronal phenotypes have been well-defined^9, 14^.

The aim of this study was to test the hypothesis that blockade of Cx43 hemichannels with a mimetic peptide would selectively improve survival of striatal phenotypic neurons after global cerebral ischaemia in term-equivalent fetal sheep at 0.85 of gestation. Further, we tested the secondary hypothesis that improved survival of GABA-ergic striatal neurons would be associated with reduced seizure burden, faster rate of return to sleep state cycling and recovery of EEG activity. At this gestational age, brain maturation is equivalent to the human infant at term^15, 16^.

## Results

**Figure 1:**
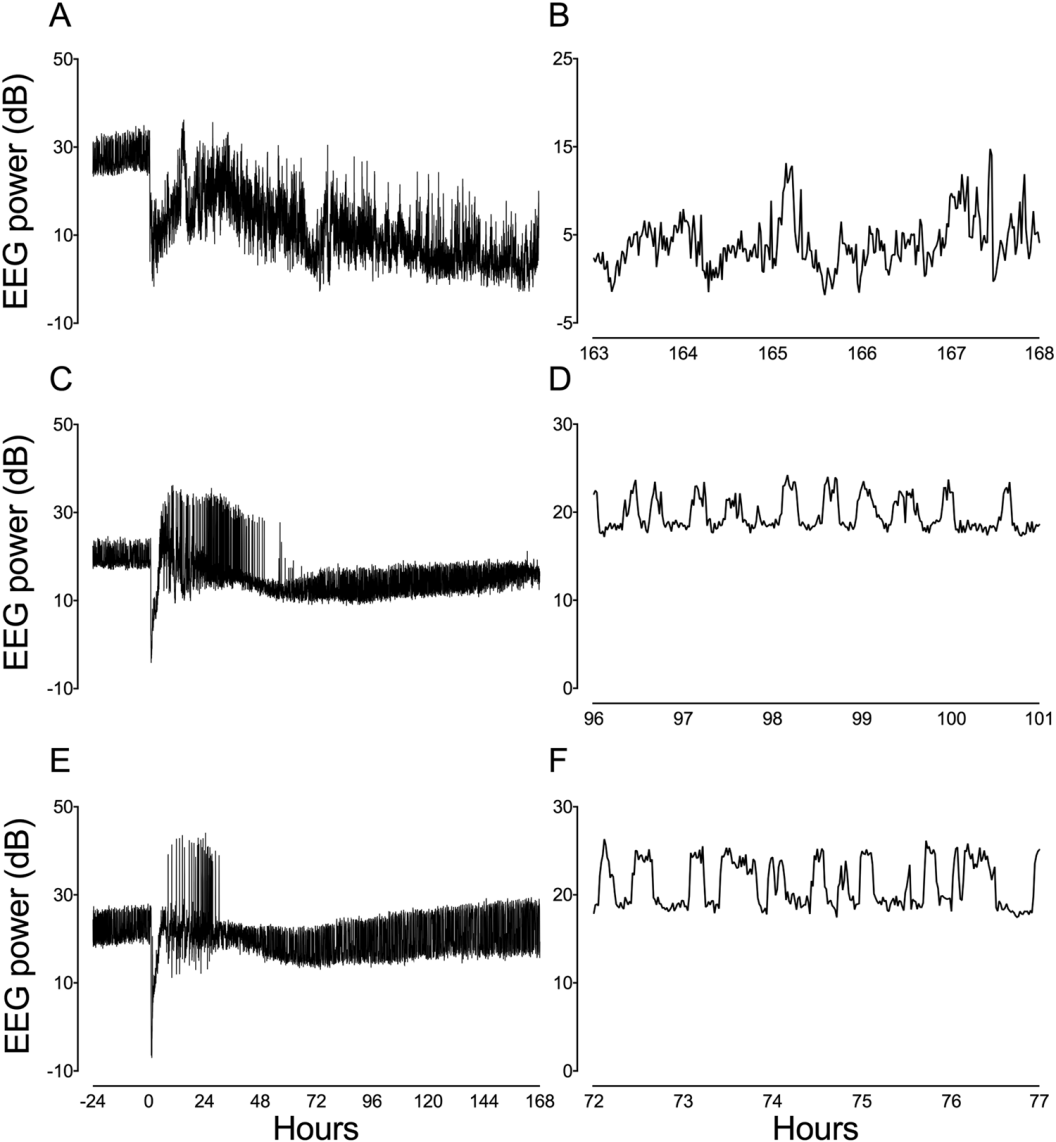
Representative continuous EEG and sleep state cycling data (1 min averages) from a fetus in the ischaemia + vehicle group (**A,B**). Note the continued suppression of EEG power after asphyxia (0 h) followed by a large prolonged rise in EEG activity between 10 and 168 h, reflecting electrographic seizure activity, and no sleep state cycling by 168 h (7 d). In the examples from a fetus in the ischaemia + short infusion (**C,D**) and ischaemia + long infusion groups (**E,F**), the pattern of seizure activity was much more discrete than after ischaemia + vehicle and sleep state cycling returned by 96 and 72 h (days 4 and 3), respectively.

Fetal arterial blood gases, pH, glucose and lactate, and baseline EEG power did not differ between groups and were within the normal range by our laboratory’s standards^10^. During ischaemia, there was rapid suppression of EEG power that did not differ between groups (Table 1 and Fig. 1).

**Table 1:** Neurophysiological data during ischaemia and recovery in the ischaemia + vehicle, ischaemia + short infusion and ischaemia + long infusion groups.

	Ischaemia + vehicle	Ischaemia + short infusion	Ischaemia + long infusion
ΔEEG power during ischaemia (dB)	−14.2 ± 2.5	−19.1 ± 2.7	−15.8 ± 6.2
Total seizure burden (min)	755 ± 308	357 ± 312*	259 ± 279*
Sleep state cycling (day of return)	6 ± 1	4 ± 1*	3 ± 1*
7 d EEG power (dB)	−12.7 ± 5.7	−8.8 ± 3.9	−4.4 ± 3.8#

Data are mean ± SD. *P < 0.05 vs. ischaemia + vehicle, ^#^P < 0.05 vs. ischaemia + short infusion.

### Recovery

In the ischaemia + vehicle and ischaemia + peptide groups, EEG power remained suppressed for approximately 8 h after ischaemia before transiently increasing to near baseline levels, in parallel with the period of secondary seizure activity. There was no difference in neurophysiological recovery between the ischaemia groups in this interval. From 2 d of recovery, secondary suppression of EEG power was observed in the ischaemia + vehicle group that lasted until the end of the experiment. Time sequence changes in neurophysiological data throughout the recovery period have been reported in Davidson *et al*.^10^. Total seizure burden was reduced in the ischaemia + short and ischaemia + long infusion groups compared to ischaemia + vehicle (Table 1 and Fig. 1). The rate of recovery of sleep state cycling was faster in the ischaemia + short and ischaemia + long infusion groups compared to ischaemia + vehicle (Table 1 and Fig. 1). Seven days after cerebral ischaemia, EEG power was not significantly different in the ischaemia + short infusion group from ischaemia + vehicle, but was significantly improved in the ischaemia + long infusion group (Table 1 and Fig. 1).

### Histopathology

**Figure 2:**
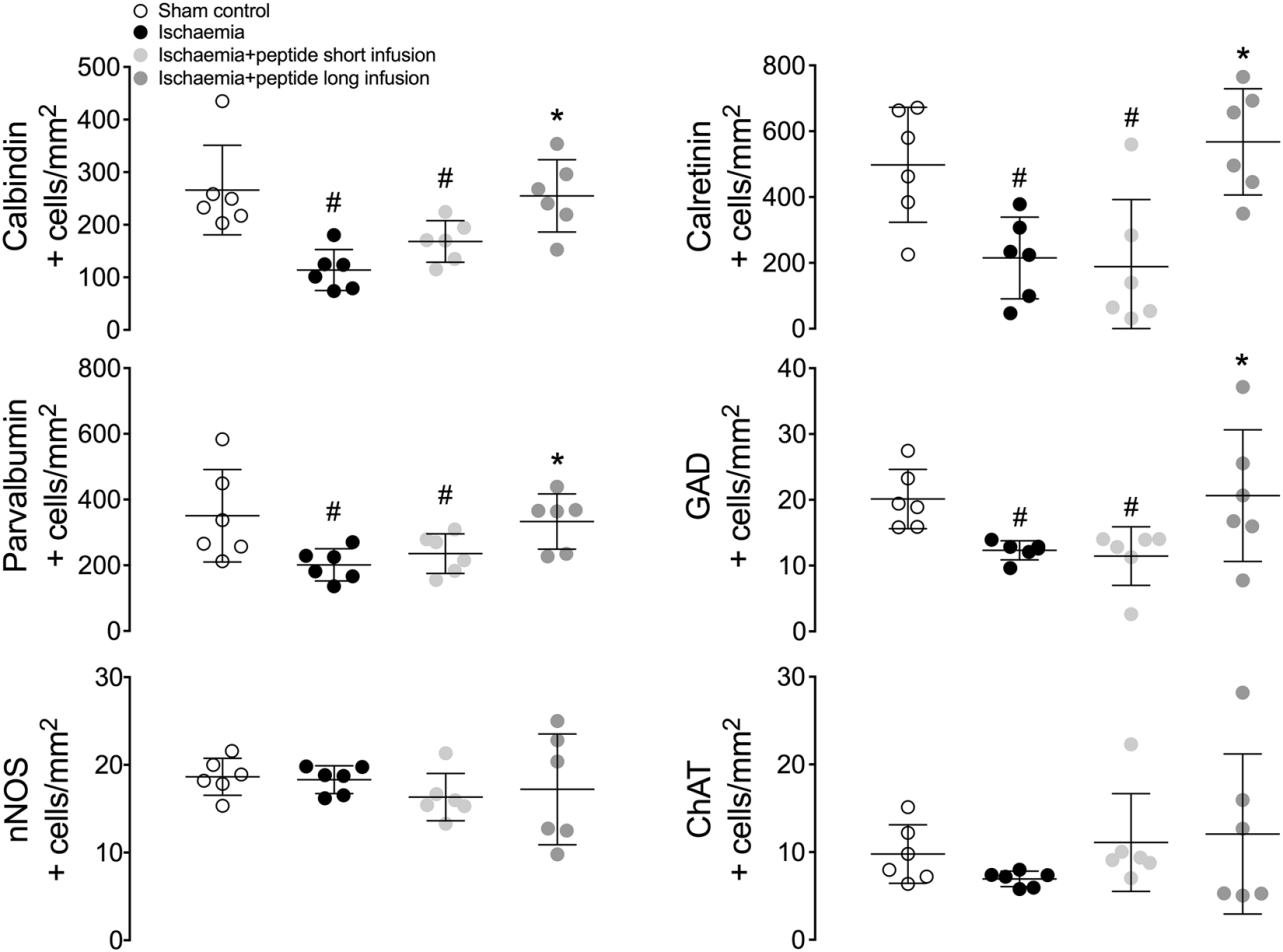
Phenotypic striatal neuronal density in the striatum (including the caudate nucleus and putamen). Data are mean ± SD from n = 6/group. ^#^p < 0.05 vs. sham ischaemia; *P < 0.05 vs. ischaemia + vehicle.

**Figure 3:**
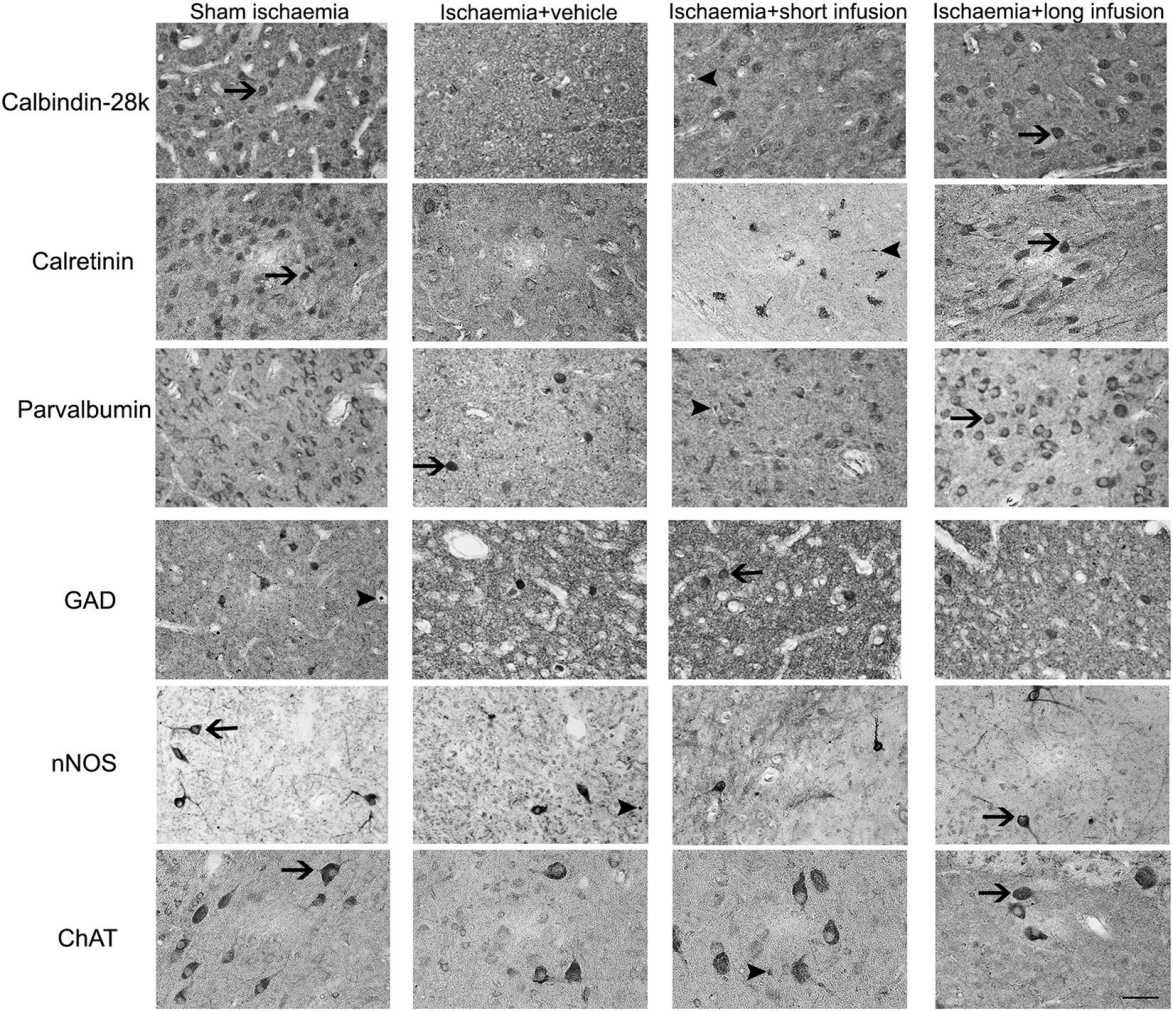
Representative photomicrographs of striatal phenotypic neurons in the caudate nucleus from sham ischaemia, ischaemia + vehicle, ischaemia + short infusion and ischaemia + long infusion groups at 40x magnification. Arrowheads with tail show examples of neurons that were counted, arrowheads show examples of neurons that were not counted. Scale bar is 200 µm.

**Figure 4:**
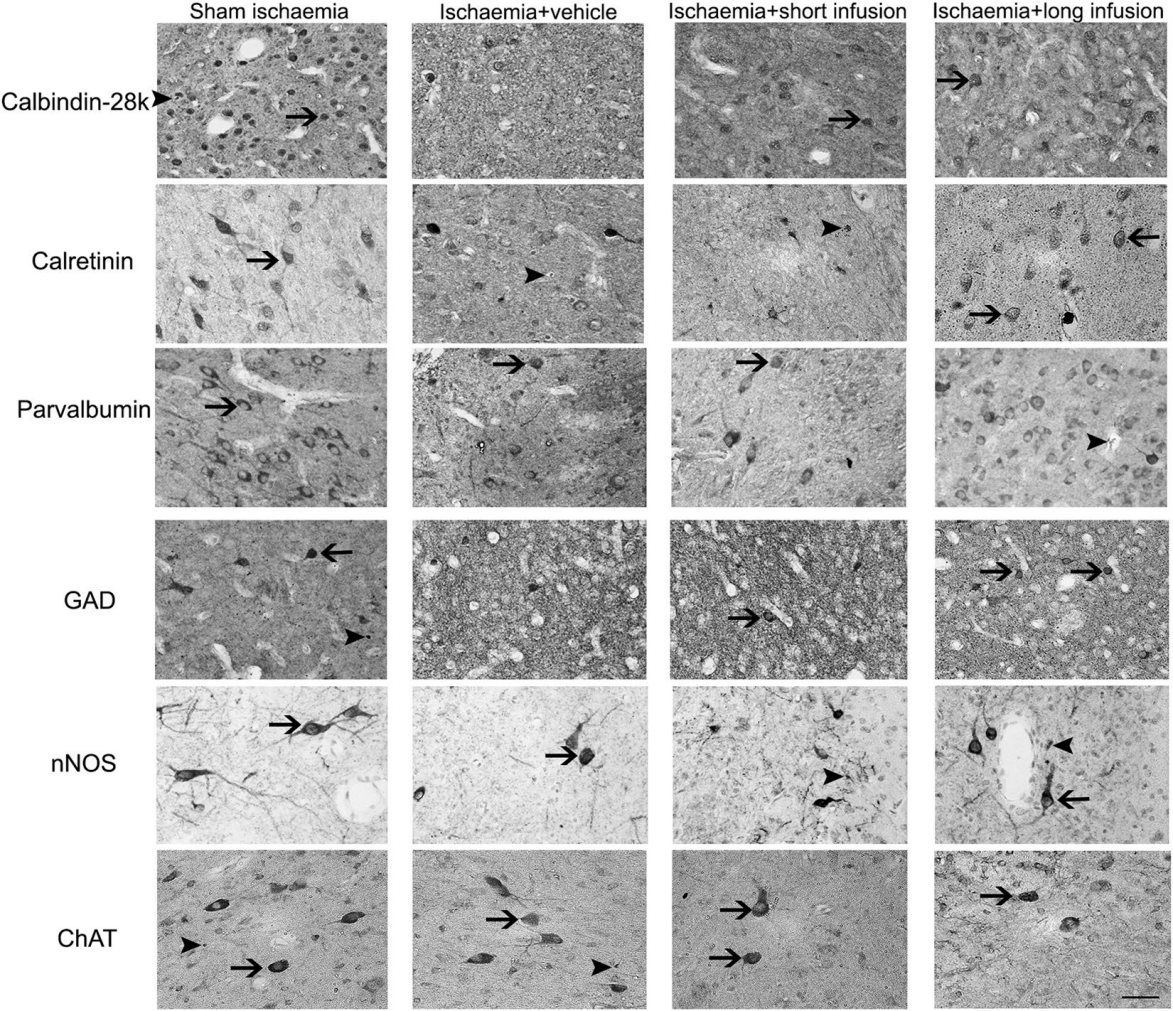
Representative photomicrographs of striatal phenotypic neurons in the putamen from sham ischaemia, ischaemia + vehicle, ischaemia + short infusion and ischaemia + long infusion groups at 40x magnification. Arrowheads with tail show examples of neurons that were counted, arrowheads show examples of neurons that were not counted. Scale bar is 200 µm.

Ischaemia + vehicle was associated with loss of calbindin-28k, calretinin, parvalbumin and GAD positive neurons compared to sham ischaemia (P < 0.05; Figs 2–4). After ischaemia, short infusion of peptide did not improve survival of striatal neurons (P < 0.05 vs. sham ischaemia). Long infusion of peptide was associated with improved survival of calbindin-28k, calretinin, parvalbumin and GAD positive striatal neurons (P < 0.05 vs. ischaemia + vehicle). Ischaemia was not associated with significant loss of nNOS or ChAT positive neurons after 7 days of recovery.

### Correlations

**Figure 5:**
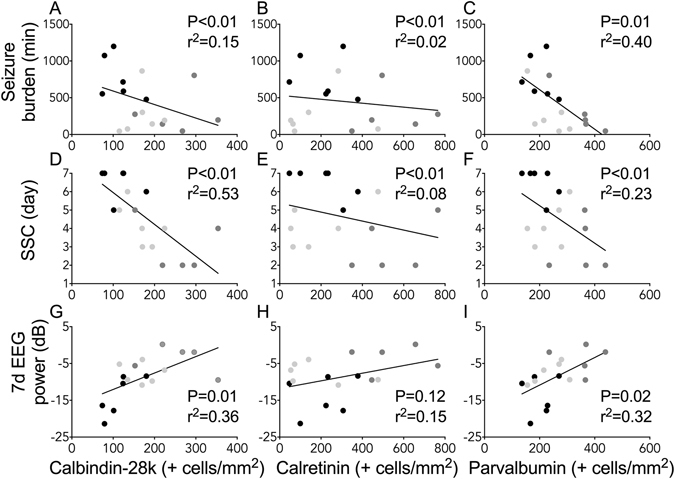
Correlations between neurophysiological outcomes and histopathology in ischaemia + vehicle, n = 6; ischaemia + short infusion, n = 6 and ischaemia + long infusion, n = 5 (neurophysiological data were not available from 1 subject in the ischaemia + long infusion group). Panels (**A–C**) Correlations between total seizure burden and survival of striatal calbindin-28k, calretinin and parvalbumin positive neurons. (**D–F**) Correlations between the day that sleep state cycling (SSC) resumed after ischaemia and survival of striatal calbindin-28k, calretinin and parvalbumin positive neurons. (**G–I**) Correlations between EEG power on day 7 of recovery and survival of striatal calbindin-28k, calretinin and parvalbumin positive neurons.

Electrographic seizure burden was inversely related to improved survival of calbindin-28k (non-linear regression: P < 0.01, r^2^ = 0.15; Fig. 5), calretinin (linear regression: P < 0.01, r^2^ = 0.02) and parvalbumin positive striatal neurons (linear regression: P = 0.01, r^2^ = 0.40). The day of return of sleep state cycling was inversely related to improved survival of calbindin-28k (linear regression: P < 0.01, r^2^ = 0.53; Fig. 5), calretinin (non-linear regression: P < 0.01, r^2^ = 0.08) and parvalbumin positive striatal neurons (non-linear regression: P < 0.01, r^2^ = 0.23). Recovery of EEG power after 7 days was positively associated with improved survival of calbindin-28k (linear regression: P = 0.01, r^2^ = 0.36; Fig. 5) and parvalbumin positive striatal neurons (linear regression: P = 0.02, r^2^ = 0.32). There was no significant correlation between recovery of EEG power and survival of calretinin, nNOS or ChAT positive striatal neurons.

## Discussion

This study demonstrates that Cx43 hemichannel blockade, starting 90 minutes after global cerebral ischaemia and continued for 24 hours, significantly improved survival of striatal GABA-ergic neurons in term equivalent fetal sheep. Increased survival of GABA-ergic striatal neurons was associated with reduced seizure burden, faster recovery of sleep state cycling and improved recovery of EEG power after 7 days.

Basal ganglia injury at term is strongly associated with poor neurodevelopmental outcomes that include learning disability, epilepsy and cerebral palsy^1^. We have recently demonstrated that opening of connexin hemichannels, the constituents of gap junctions, plays an integral role in the early evolution of HIE^10–12^. Gap junctions are intercellular channels that link the cytoplasm of neighbouring cells to permit the exchange of small molecules and ions. Under normal conditions, gap junctions function in an open state, but undocked connexin hemichannels remain closed^17^. However, after ischaemia, oxygen glucose deprivation, metabolic inhibition or low extracellular calcium ion levels, unopposed connexin hemichannels can open^10, 18–21^, leading to disruption of resting membrane potential, extracellular release of cytotoxic levels of glutamate^22^ and adenosine triphosphate (ATP)^23^, and excessive cell swelling and rupture^24, 25^. We have previously shown that Cx43 hemichannel blockade started 90 minutes after cerebral ischaemia or asphyxia and continued for at least 25 hours markedly reduced status epilepticus, improved recovery of brain activity and reduced oligodendrocyte loss^10–12^. While there are multiple connexin hemichannel isoforms, there is strong evidence that Cx43 is the predominant astrocytic hemichannel isoform involved in the pathogenesis of neuronal injury and is one of the key therapeutic targets for research into neuroprotection^26, 27^. Additionally, it has recently been reported that extracellular loop homology is distinct between Cx43 (Peptide 5) and other connexin isoforms at amino-acid position 5 (leucine)^28^. This is a highly sensitive position where a single amino-acid substitution causes complete loss of function^28^. This suggests that the Cx43 mimetic peptide is isoform specific and is unlikely to be effective against other connexin isoforms found on neuronal, vascular or glial cells.

In the present study, cerebral ischaemia was associated with reduced survival of cabindin-28k, calretinin, parvalbumin and GAD positive neurons after 7 days of recovery. However, there was no effect of ischaemia on nNOS or ChAT positive neurons after 7 days. These findings are broadly consistent with observations after shorter periods of recovery from asphyxia or ischaemia in preterm and term-equivalent fetal sheep^9, 14, 29, 30^. Collectively, these data suggest that the GABA-ergic striatal neurons, particularly those expressing the intracellular calcium binding proteins: calbindin-28k, calretinin and parvalbumin, are highly susceptible to ischaemic injury in the term-equivalent fetus.

The vulnerability of GABA-ergic neurons that express calcium binding proteins may reflect greater dependency of these neurons on their calcium buffering system for homeostasis, such that impaired calcium buffering capacity during and after HI may underlie their susceptibility to injury. Furthermore, we did not observe significant neuronal injury in striatal neurons that lacked calcium binding proteins, such as those expressing nNOS and ChAT. These data further support the idea that neurons expressing calcium binding proteins show greater vulnerability to ischaemic injury. Critically, excessive intracellular calcium accumulation has been implicated in the pathogenesis of mitochondrial dysfunction and neuronal apoptosis and necrosis in the adult^31, 32^ and developing brain^33^. Furthermore, striatal mitochondria show greater sensitivity to disturbances in calcium homeostasis compared to mitochondria from cortical and hippocampal neurons^34^. Collectively, these data suggest that developing striatal GABA-ergic neurons are susceptible to increased extracellular glutamate after HI, possibly due to greater susceptibility to disturbances in calcium homeostasis and subsequent mitochondrial dysfunction.

Alternatively, GABA-ergic neurons may be particularly susceptible to ATP released into the extracellular space from open astrocytic connexin hemichannels after HI. Glia and striatal GABA-ergic neurons express purinergic receptors^35, 36^. In peritraumatic areas, extracellular ATP can activate neuronal and glial purinergic receptors and induce cellular injury by promoting extracellular calcium influx through the receptor channel^37^, as well as augmenting the pro-inflammatory function of activated microglia^38, 39^. Furthermore, activation of astrocytic purinergic (P2Y) receptors by extracellular ATP has been shown to increase intracellular calcium accumulation, which enhances connexin hemichannel opening and leads to ‘ATP-induced ATP release’^40, 41^, increasing the potential for ATP-mediated neurotoxicity. Taken with the finding discussed next that prolonged connexin hemichannel blockade after ischaemia was selectively protective, these data strongly support a key role for ATP in the pathogenesis of basal ganglia injury in the developing brain.

The short infusion of the mimetic peptide was not associated with improved survival of striatal neurons. In contrast, a long infusion for 25 hours was associated with improved survival of calbindin-28k, calretinin, parvalbumin and GAD positive GABA-ergic neurons. Critically, GABA-ergic neurons, particularly those expressing calbindin-28k, calretinin and parvalbumin calcium binding proteins make up the majority of the striatal neural population^42, 43^. Thus, these data demonstrate that prolonged connexin hemichannel blockade markedly improved the overall survival of striatal neurons. The improved survival of striatal GABA-ergic neurons observed in this study was similar to previous reports that have shown improved striatal GABA-ergic neuronal survival after inhibition of neuronal nitric oxide synthase^14^ and therapeutic hypothermia^29^ after cerebral HI. Conversely, supplementation with low dose recombinant insulin-like growth factor-1 improved survival of GAD-positive striatal neurons but did not improve survival of parvalbumin-positive cells^9^. Based on the likely cellular mechanisms responsible for neuroprotection in these studies and ours, we can reasonably suggest that post-insult production of reactive nitrogen species, reduced trophic support, excessive purinergic receptor activation, secondary inflammation and intracellular accumulation of excitatory amino acids and calcium are potentially key pathophysiological mechanisms involved in basal ganglia injury and should be the subject of further investigation.

Immunoreactive ChAT and nNOS interneurons were relatively resistant to ischaemic injury in the present study. In adult rats, cholinergic interneurons were more tolerant to glutamate receptor mediated excitotoxicity than spiny projection neurons, possibly due to their lack of the glutamate receptor sub-units: GluR1, GluR2/3 and GluR4^44^. Resistance of striatal cholinergic interneurons to injury may be partially mediated by their increased expression of the free radical scavenger superoxide dismutase^45, 46^. Furthermore, striatal GABA-ergic interneurons containing nNOS have previously been shown to be resistant to hypoxic ischaemic injury^47, 48^ and neurodegeneration in Huntington’s and Alzheimer’s disease patients^49, 50^. While the exact mechanisms of their resistance to injury is unclear, it may include a nitric oxide mediated reduction of N-methyl-D-aspartate-mediated influx of calcium into the intracellular space and/or increased regional blood flow mediated by local nitric oxide release^47^.

We observed improved recovery of EEG power in fetuses treated with the long infusion of peptide. In line with this, a positive relationship between improved recovery of EEG power at 7 days and survival of striatal calbindin-28k and parvalbumin positive neurons was observed. This is consistent with the clinical finding in term infants that improved recovery of EEG power is strongly associated with better neurodevelopmental outcome^51–53^, reflecting reduced neural injury. These data suggest that improved survival of striatal GABA-ergic neurons, in addition to partial preservation of cortical neurons, as previously reported in Davidson *et al*.^10^, contribute to improved recovery of EEG activity after HI.

Seizures are common after HI^54, 55^, and are strongly associated with poor neurodevelopmental outcomes^56–58^. Compared to adults, the neonatal brain is susceptible to electrographic seizures, possibly related to peak synaptic and dendritic spine density and/or relative abundance of glutamatergic (pro-excitatory) neurons^59^. During seizures, blood flow to the basal ganglia is increased compared to the cortex, cerebellum or brainstem, suggesting increased local electrical activity and metabolic demand^60^. Furthermore, in term infants, seizures were associated with an increase in lactate:choline within the basal ganglia, suggesting that seizures may independently increase local metabolic stress^57^. In this study peptide infusion was associated with a reduction in electrographic seizures, with the greatest effect in the long infusion group. We observed an inverse relationship between electrographic seizure burden and numbers of surviving calbindin-28k, calretinin and parvalbumin positive striatal neurons; parvalbumin positive neurons showed the strongest correlation with reduced seizure burden. This raises the possibility that loss of GABA-ergic interneurons within the basal ganglia, particularly those expressing the parvalbumin protein, facilitates disinhibition and thereby promotes seizure propagation. Supporting this, parvalbumin deficient mice are significantly more susceptible to pentylenetetrazole-induced seizures than controls^61^. Alternatively, it may reflect a differential susceptibility of GABA-ergic neurons to seizures, such that neurons expressing the parvalbumin protein are more susceptible to seizure-induced metabolic stress.

Striatal lesions have been associated with destabilisation of sleep state architecture and impaired electrocortical arousal^62^. Furthermore, striatal medium spiny neurons generate distinct firing patterns during the sleep-state cycle, mainly characterised by brisk rhythmic neuronal firing during non-rapid eye movement sleep due to fluctuations between hyperpolarised quiescence and a depolarised state^63^. These data suggest that basal ganglia neurons, particularly medium spiny neurons, contribute to the neural circuitry that controls sleep state. Consistent with this, improved survival of striatal GABA-ergic neurons, particularly the calbindin-28k medium spiny neurons, was highly associated with faster return of sleep state cycling.

Intriguingly, peptide infusion was associated with a graded improvement in neurophysiological recovery, such that short infusion was associated with an intermediate reduction in electrographic seizure burden and improvement in the rate of return to sleep state cycling. We have previously shown that short infusion of Peptide 5 was associated with improved recovery of EEG power after cerebral ischaemia^10^. However, in the present study, short infusion of peptide was not associated with improved survival of any striatal neuronal phenotype. These data demonstrate that the relationship between neuronal loss and brain activity is complex and not purely dependent on improved cell survival. Speculatively, improved functionality of surviving neurons and glia may have contributed to the intermediate improvement in neurophysiological recovery after the short infusion.

Some limitations of the present study should be considered. It is important to reflect that correlation is of course not causation. In this study we used correlative analysis to better understand the relationship between EEG function and survival of phenotypically distinct striatal neurons and thus to highlight hypotheses that can be studied further. Further, the present study used a relatively prolonged recovery period (7 days) to allow us to test the efficacy of Cx43 hemichannel blockade on the evolution of the secondary phase of neuronal injury, which occurs for several days after the insult^56^. However, using a prolonged recovery time precluded us from examining specific cellular mechanisms that mediated neuroprotection in this study. This is one of the limitations of performing large animal translational studies which are expensive relative to rodent studies^64^. Thus, identifying specific cellular mechanisms would require a separate series of experiments, which will be the subject of future investigations in our laboratory. Similarly, it is not possible to be certain whether the mimetic peptide acts directly on striatal Cx43 or potentially via paraventricular actions. Nevertheless, we have previously shown that propidium iodide uptake is reduced by i.c.v. peptide infusion in the parasagittal white matter and in grey matter^10^. Given that Peptide5 is a small peptide that is highly specific to the extracellular loop of Cx43^28^, this supports a local effect.

In conclusion, prolonged infusion of a mimetic peptide targeting Cx43 hemichannels was associated with improved survival of striatal GABA-ergic neurons, reduced electrographic seizure burden, faster return to sleep state cycling and improved recovery of EEG power 7 days after HI. These data suggest that connexin hemichannel blockade has potential to reduce the severity of basal ganglia injury in term infants with HIE.

## Methods

All procedures were approved by the Animal Ethics Committee of The University of Auckland following the New Zealand Animal Welfare Act, and the Code of Ethical Conduct for animals in research established by the Ministry of Primary Industries, Government of New Zealand. Romney Suffolk fetal sheep underwent aseptic surgery between 118 and 124 days gestation (term = 147 days). Food but not water was withdrawn 18 h before surgery. Ewes were given long acting oxytetracycline (20 mg/kg, Phoenix Pharm, Auckland, New Zealand) intramuscularly 30 minutes before the start of surgery. Anaesthesia was induced by intravenous injection of propofol (5 mg/kg; AstraZeneca Limited, Auckland, New Zealand) and maintained using 2–3% isoflurane in O_2_ (Bomac Animal Health, NSW, Australia). During surgery, ewes received an i.v. infusion of isotonic saline (250 mL/h) to maintain fluid balance. Depth of anaesthesia, maternal heart rate and respiration were continuously monitored by trained anaesthetic staff.

### Instrumentation

Instrumentation of fetuses has been previously described^10^. In brief, following a maternal midline abdominal incision, the fetus was exposed, and polyvinyl catheters were inserted in the right and left brachial arteries and amniotic cavity. The vertebral-occipital anastomoses were ligated and a pair of inflatable carotid artery occluder cuffs was placed around both carotid arteries^65, 66^. Two pairs of electroencephalograph (EEG) electrodes (AS633-7SSF; Cooner Wire, Chatsworth, CA, USA) were placed through burr holes onto the dura over the parasagittal parietal cortex (10 and 20 mm anterior to bregma and 10 mm lateral) and secured with cyanoacrylate glue. A reference electrode was sewn over the occiput. An intracerebroventricular (ICV) catheter was placed into the left lateral ventricle (6 mm anterior and 4 mm lateral to bregma; measured dead space, 0.70 ± 0.02 mL). Accurate placement of the ICV catheter was shown during the surgical procedure by confirming that cerebrospinal fluid could flow freely out of and into the lateral ventricle. Further, we have previously shown that this was associated with accurate placement of the ICV catheter (~4 days after surgery) in a pilot study, whereby fluorescent peptide was infused into the left lateral ventricle from 90 min until 6 hours after ischaemia. Inspection of freshly cut coronal sections revealed a high concentration of peptide surrounding the left lateral ventricles with widespread diffusion into subcortical and cortical areas^10^.

Antibiotics (gentamicin; 80 mg; Roussel Ltd., Auckland, New Zealand) were administered into the amniotic sac before closing the uterus. A maternal long saphenous vein was implanted to provide access for postoperative care. Sheep were housed in separate metabolic cages with access to water and food *ad libitum* in a temperature-controlled room (16 ± 1 °C, humidity 50 ± 10%) with a 12 h light dark cycle. Ewes received intravenous antibiotics daily for 4 days (benzylpenicillin sodium; 600 mg; Novartis, Auckland, New Zealand and gentamycin; 80 mg). Fetal catheters were maintained patent by continuous infusion with heparinised saline (20 IU/mL), and the maternal catheter was maintained patent by daily flushing.

### Recordings

Continuous fetal EEG recordings began 1 d before and continued until 7 d after bilateral carotid artery occlusion. The analogue fetal EEG signal was low pass filtered with a cut off frequency set with the −3 dB point at 30 Hz, and digitised at a sampling rate of 512 Hz. EEG power was derived from the power spectrum signal between 0.5 and 20 Hz^67^ and was normalised by log transformation (dB, 20 × log power).

### Experimental protocol

Experiments began at 128 ± 1 (mean ± SD) days gestation. Global cerebral ischaemia was induced by reversible inflation of the carotid artery occluder cuffs with saline for 30 minutes. Successful occlusion was confirmed by rapid sustained suppression of EEG activity. Ninety minutes after the end of ischaemia, fetuses were randomly assigned to receive an ICV infusion of a mimetic peptide that blocks the extracellular loop of Cx43 (Peptide 5, amino acid sequence: H-Val-Asp-Cys-Phe-Leu-Ser-Arg-Pro-Thr-Glu-Lys-Thr-OH^13, 28^) as either a short infusion; 50 µmol/kg, dissolved in 1 mL of artificial cerebrospinal fluid and infused over 1 h (n = 6), or a long infusion; 50 µmol/kg over 1 h then 50 µmol/kg over 24 h (n = 6) at a rate of 1 mL over the first hour then 1 mL over 24 h. Controls received either sham ischaemia (n = 6) or cerebral ischaemia (n = 6) followed by an equivalent ICV infusion of vehicle (artificial cerebrospinal fluid: KCl 5 mmol/L, NaCl 137 mmol/L, CaCl_2_ 0.8 mmol/L, 0.1% bovine serum albumin, pH 7.4). The ICV infusion was delivered using an external infusion pump (SS-2222, Harvard Apparatus, Holliston, MA, USA). For all groups, the dead-space in the ICV cannula was cleared by preinfusing 0.7 ml of artificial CSF at 1 ml/h, before the start of the peptide or vehicle infusion. Randomisation was stratified by cohort to control for the time of year and breeding season to avoid any potential confounding related to intra-or inter-seasonal differences in fetal growth due to changes in flock management, nutrition, climate or paternal genotype^68^. Ewes and fetuses were killed 7 days after ischaemia with an overdose of sodium pentobarbitone (9 g intravenous to the ewe; Pentobarb 300; Chemstock, Christchurch, New Zealand).

### Histopathology

At post mortem, the fetal brains were fixed *in situ* via gravity perfusion with 500 mL of 0.9% saline then 1 L of 10% phosphate buffered formalin from a height of 1 m. Following removal from the skull, brains were fixed for a further 5 days before being processed and embedded using a standard paraffin tissue preparation. Coronal brain slices were cut (10 μm thick) using a microtome (Leica Jung RM2035, Leica Microsystems, Albany, New Zealand). Brain regions of the forebrain used for analysis included sections taken at the level of the mid striatum, 26 mm anterior to stereotaxic zero according to the fetal sheep stereological atlas^69^. Slides were dewaxed in xylene and rehydrated in decreasing concentrations of ethanol and then washed in 0.1 mol/L phosphate buffered saline (PBS). Antigen retrieval was performed in citrate buffer using the pressure cooker technique in an antigen retrieval system (EMS Antigen 200 Retriever, Emgrid, Australia). Endogenous peroxidase quenching was performed by incubation in 0.1% H_2_O_2_ in methanol. Non-specific antigens were blocked using 3% normal goat serum. The sections were labelled with 1:200 rabbit anti-Calbindin 28k (medium sized GABA-ergic spiny projection neurons; Swant® Ltd. Rte de l′Ancienne Papeterie, Marly, Switzerland), 1:500 rabbit anti-neuronal nitric oxide synthase (nNOS, medium sized GABA-ergic aspiny interneurons; Abcam, via Sapphire Bioscience, Hamilton, NZ), 1:200 rabbit anti-glutamic acid decarboxylase (GAD 67 and 65 positive, medium sized GABA-ergic aspiny interneurons; Abcam), 1:200 rabbit anti-Calretinin (medium to large GABA-ergic aspiny interneurons; Swant®), 1:200 rabbit anti-parvalbumin (medium to large GABA-ergic aspiny interneurons Swant®) and 1:200 mouse anti-cholinergic acetyltransferase (ChAT, large cholinergic aspiny interneurons; Abacus ALS. Auckland, NZ) overnight at 4 °C. Sections were incubated in biotin conjugated IgG (1:200, goat anti rabbit or mouse; Vector Laboratories, Burlingame, USA) for 3 h at room temperature. Sections were incubated in ExtrAvidin®-Peroxidase (1:200, Sigma-Aldrich, Auckland, New Zealand) for 2 h at room temperature and allowed to react with 3,3′-diaminobenzidine tetrahydrochloride (Sigma Aldrich). The reaction was stopped by washing in PBS before sections were dehydrated and mounted.

**Figure 6:**
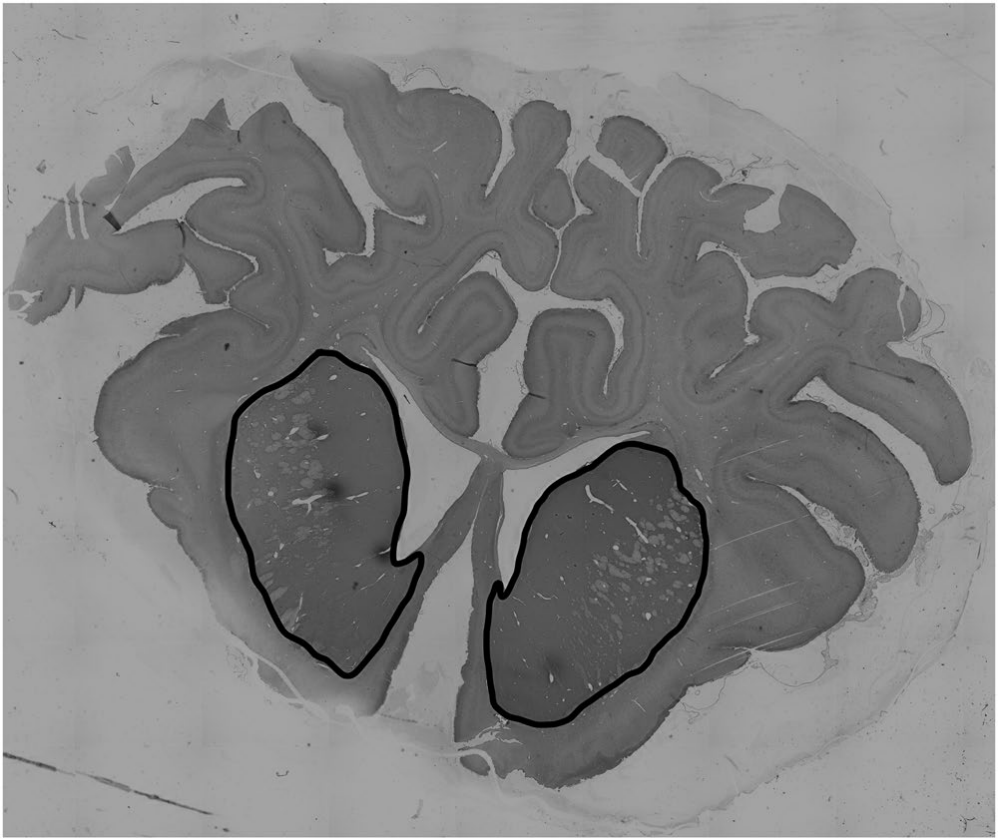
Photomicrograph showing the striatal area that was outlined at 2x and randomly sampled at 20x magnification, using a sampling grid set at 2200 × 2200. Striatal phenotypic neuronal populations were quantified from each hemisphere across 2 sections.

Estimates of striatal neuron phenotype density were quantified by light microscopy using Stereoinvestigator software (Microbrightfield Bioscience (MBF), Williston, VT, USA). The striatum, including the caudate nucleus and putamen, was outlined at 2x magnification (Fig. 6) and neurons were quantified at 20x magnification. A sampling grid of 2200 × 2200 µm was placed at random rotation over the outlined area. Positively stained cells were counted within a 400 × 400 µm counting frame; cells touching the top and left-hand corners of the frame were excluded. Neurons were counted if they were morphologically normal; cells displaying condensed or fragmented nuclei were not counted^70^. When the investigator (RG) was unsure whether a neuron was morphologically normal, 40x magnification was used to confirm whether the cell should be excluded from the count. Average scores from both hemispheres from two sections were calculated for each subject.

### Data analysis

Off-line physiological data analysis was performed using LabVIEW based customised programs (LabVIEW for Windows, National Instruments Inc.). Seizures were identified visually and defined as sudden repetitive and evolving waveforms in the EEG signal lasting more than 10 seconds and of an amplitude greater than 20 μV^71^. Sleep state cycling was defined as a repetitive alternating pattern of high and low voltage EEG activity with each phase lasting approximately 20 minutes. All neurophysiological assessments and neuronal counts were performed by an assessor who was blinded to the treatment groups.

Statistical analysis was undertaken using SPSS (v22 SPSS, Chicago, IL, USA) and GraphPad software (v6.0 GraphPad software, La Jolla, CA, USA). Between groups comparison was performed using a one-way ANOVA with Fisher’s LSD test for multiple comparisons. Linear and non-linear regressions were used as appropriate to compare the relationship between EEG recovery (including EEG power on day 7 of recovery, sleep state cycling and total seizure burden) and survival of striatal phenotypic neurons. Statistical significance was accepted when P < 0.05. Data are means ± SD.
